# 4-Formyl-2-nitro­phenyl 2-chloro­benzoate

**DOI:** 10.1107/S1600536813031346

**Published:** 2013-11-23

**Authors:** Rodolfo Moreno-Fuquen, Geraldine Hernandez, Javier Ellena, Carlos A. De Simone, Juan C. Tenorio

**Affiliations:** a Departamento de Química, Facultad de Ciencias, Universidad del Valle, Apartado 25360, Santiago de Cali, Colombia; b Instituto de Física de São Carlos, IFSC, Universidade de São Paulo, USP, São Carlos, SP, Brazil

## Abstract

In the title compound, C_14_H_8_ClNO_5_, the benzene rings form a dihedral angle of 19.55 (9)°. The mean plane of the central ester group [r.m.s. deviation = 0.024 Å] forms dihedral angles of 53.28 (13) and 36.93 (16)°, respectively, with the nitro- and chloro-substituted rings. The nitro group forms a dihedral angle of 19.24 (19)° with the benzene ring to which it is attached. In the crystal, mol­ecules are linked by weak C—H⋯O hydrogen bonds, forming *C*(7) chains, which run along [100].

## Related literature

For industrial applications of nitro­aromatic compounds, see: Ju & Parales (2010 ▶). For similar structures, see: Moreno-Fuquen *et al.* (2013*a* ▶,*b* ▶); For information on hydrogen bonds, see: Nardelli (1995 ▶). For hydrogen-bond graph-set motifs, see: Etter (1990 ▶).
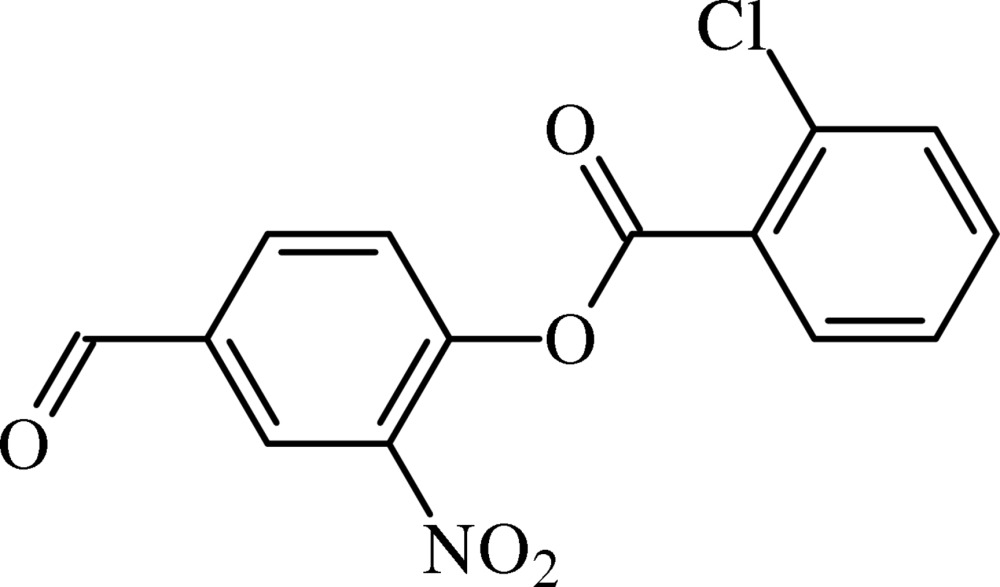


## Experimental

### 

#### Crystal data

C_14_H_8_ClNO_5_
*M* *_r_* = 305.66
Orthorhombic, 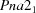

*a* = 16.2367 (7) Å
*b* = 7.1047 (2) Å
*c* = 11.4018 (3) Å
*V* = 1315.28 (8) Å^3^
*Z* = 4
Mo *K*α radiation
μ = 0.31 mm^−1^
*T* = 293 K
0.22 × 0.19 × 0.03 mm

#### Data collection

Nonius KappaCCD diffractometer
4581 measured reflections
2449 independent reflections
1762 reflections with *I* > 2σ(*I*)
*R*_int_ = 0.030

#### Refinement

*R*[*F* ^2^ > 2σ(*F* ^2^)] = 0.044
*wR*(*F* ^2^) = 0.120
*S* = 1.03
2449 reflections
194 parameters
1 restraint
H atoms treated by a mixture of independent and constrained refinement
Δρ_max_ = 0.20 e Å^−3^
Δρ_min_ = −0.19 e Å^−3^
Absolute structure: Flack parameter determined using 664 quotients [(*I* ^+^)−(*I* ^−^)]/[(*I* ^+^)+(*I* ^−^)] (Parsons *et al.*, 2013 ▶)
Absolute structure parameter: −0.05 (5)

### 

Data collection: *COLLECT* (Nonius, 2000 ▶); cell refinement: *SCALEPACK* (Otwinowski & Minor, 1997 ▶); data reduction: *DENZO* (Otwinowski & Minor, 1997 ▶) and *SCALEPACK*; program(s) used to solve structure: *SHELXS97* (Sheldrick, 2008 ▶); program(s) used to refine structure: *SHELXL2013* (Sheldrick, 2008 ▶); molecular graphics: *ORTEP-3 for Windows* (Farrugia, 2012 ▶) and *Mercury* (Macrae *et al.*, 2006 ▶); software used to prepare material for publication: *WinGX* (Farrugia, 2012 ▶).

## Supplementary Material

Crystal structure: contains datablock(s) I, global. DOI: 10.1107/S1600536813031346/lh5661sup1.cif

Structure factors: contains datablock(s) I. DOI: 10.1107/S1600536813031346/lh5661Isup2.hkl

Supplementary material file. DOI: 10.1107/S1600536813031346/lh5661Isup3.cml

Additional supplementary materials:  crystallographic information; 3D view; checkCIF report

## Figures and Tables

**Table 1:** Hydrogen-bond geometry (Å, °)

*D*—H⋯*A*	*D*—H	H⋯*A*	*D*⋯*A*	*D*—H⋯*A*
C14—H14⋯O4^i^	1.10 (6)	2.48 (6)	3.381 (7)	138 (4)

Symmetry code: (i) 
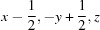
.

**Fig. 1.**
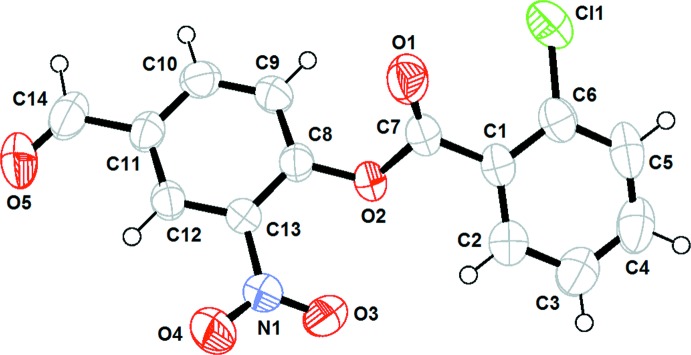
The molecular structure of the title compound with displacement ellipsoids drawn at the 50% probability level. H atoms are shown as spheres of arbitrary radius.

**Fig. 2.**
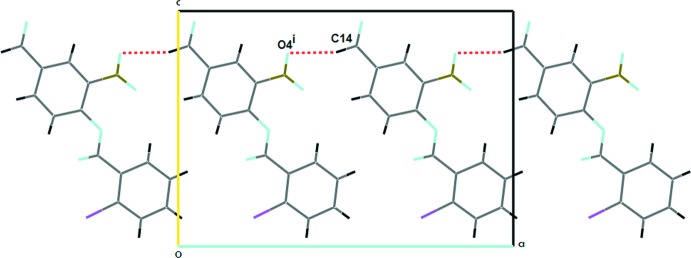
Part of the crystal structure of (I), showing the formation of chains running along [100] (symmetry code: (i) x-1/2, -y+1/2, z).

## supplementary crystallographic information

### 1. Comment

The title compound (I) is part of a series of studies on the structural properties of formyl nitro aryl benzoates, and extends from earlier work from our research group with the synthesis of 4-formyl-2-nitrophenyl 4-bromo benzoate (FBrB) (Moreno-Fuquen *et al.*, 2013*a*). The nitroaromatic compounds and their derivatives constitute a main class of industrial chemicals and are widely used as intermediates in the synthesis of many varied products, ranging from drugs, pigments, pesticides and plant growth regulators to the explosives (Ju & Parales, 2010). The molecular structure of (I) is shown in Fig. 1. Bond lengths and bond angles of (I) correlate closely with the analogue compound (FBrB) and with other aryl benzoates reported in the literature (Moreno-Fuquen *et al.*, 2013*b*) with the exception of the dihedral angle between the benzene rings which is 19.55 (9)°. This behavior may be motivated by the presence of the chlorine atom in the ortho position thereby avoiding greater repulsion with the nitro group in the other benzene ring. The ester group (C1-C7(O1)-O2-C8) is twisted away from the nitro-substituted and chloro-substituted benzene rings by 53.28 (13)° and 36.93 (16)° respectively. The nitro group forms a dihedral angle of 19.24 (19)° with the benzene ring to which it is attached. The crystal packing shows no classical hydrogen bonds and it is stabilized by weak C—H···O intermolecular hydrogen bonds, forming C(7) chains (Etter, 1990) along [100] (see Fig. 2). The C14 atom of the aldehyde group at (x,y,z) acts as hydrogen-bond donor to O4 atom at (x-1/2,-y+1/2,+z) (see Table 1; Nardelli, 1995). This interaction probably helps to reinforce the separation between nitro and chlorine groups, which are in different rings of the molecule. In the title structure, halogen···halogen interactions are not observed.

### 2. Experimental

The reagents and solvents for the synthesis were obtained from the Aldrich Chemical Co., and were used without additional purification. The title compound was obtained through a two-step reaction. First, 2-chlorobenzoic acid (0.200 g, 1.278 mmol) was placed in reflux with thionyl chloride (5 mL) in chloroform for an hour. Then, the excess of thionyl chloride was distilled to purify the 2-chlorobenzoyl chloride obtained as a pale-yellow translucent liquid. The same reaction flask was rearranged and an equimolar solution (0.213 g) of 4-hydroxy-3-nitrobenzaldehyde in acetonitrile was dropped inside it with 0.1 mL of pyridine. The reaction mixture was taken to room temperature with constant stirring for about an hour. A colorless solid was obtained after leaving the solvent to evaporate. After some difficulties in the crystallization process giving poor quality of crystals grown in different solvents (acetonitrile, dichloromethane, acetone), a colorless crystal of good quality (with some remnant amorphous solid) was obtained from a solution of the title compound in chloroform. IR spectra were recorded on a FT-IR SHIMADZU IR-Affinity-1 spectrophotometer. Colourless crystals; m.p 386 (1)K. IR (KBr) 3427.96 cm^-1^, 3081.33 cm^-1^ (aromatic C—H); 1758.36 cm^-1^ (ester C═O); 1698.30 cm^-1^ (benzaldehyde C═O), 1216.94 cm^-1^ (ester C—O); 1533.94 cm^-1^, 1347.37 cm^-1^ (nitro –NO_2_); 1020.56 cm^-1^ (C═C); 749.93 cm^-1^(Cl—C).

### Crystal data

**Table:**

C_14_H_8_ClNO_5_	*D*_x_ = 1.544 Mg m^−^^3^
*M**_r_* = 305.66	Mo *K*α radiation, λ = 0.71073 Å
Orthorhombic, *P**n**a*2_1_	Cell parameters from 4580 reflections
*a* = 16.2367 (7) Å	θ = 3.1–26.0°
*b* = 7.1047 (2) Å	µ = 0.31 mm^−^^1^
*c* = 11.4018 (3) Å	*T* = 293 K
*V* = 1315.28 (8) Å^3^	Prism, colourless
*Z* = 4	0.22 × 0.19 × 0.03 mm
*F*(000) = 624	

### Data collection

**Table:**

Nonius KappaCCD diffractometer	*R*_int_ = 0.030
Radiation source: fine-focus sealed tube	θ_max_ = 26.0°, θ_min_ = 3.1°
CCD rotation images, thick slices scans	*h* = −20→19
4581 measured reflections	*k* = −8→8
2449 independent reflections	*l* = −14→12
1762 reflections with *I* > 2σ(*I*)	

### Refinement

**Table:**

Refinement on *F*^2^	Hydrogen site location: mixed
Least-squares matrix: full	H atoms treated by a mixture of independent and constrained refinement
*R*[*F*^2^ > 2σ(*F*^2^)] = 0.044	*w* = 1/[σ^2^(*F*_o_^2^) + (0.0719*P*)^2^] where *P* = (*F*_o_^2^ + 2*F*_c_^2^)/3
*wR*(*F*^2^) = 0.120	(Δ/σ)_max_ < 0.001
*S* = 1.03	Δρ_max_ = 0.20 e Å^−^^3^
2449 reflections	Δρ_min_ = −0.19 e Å^−^^3^
194 parameters	Absolute structure: Flack parameter determined using 664 quotients [(*I*^+^)-(*I*^-^)]/[(*I*^+^)+(*I*^-^)] (Parsons *et al.*, 2013)
1 restraint	Absolute structure parameter: −0.05 (5)

### Special details

**Table:**

Geometry. All esds (except the esd in the dihedral angle between two l.s. planes) are estimated using the full covariance matrix. The cell esds are taken into account individually in the estimation of esds in distances, angles and torsion angles; correlations between esds in cell parameters are only used when they are defined by crystal symmetry. An approximate (isotropic) treatment of cell esds is used for estimating esds involving l.s. planes.

### Fractional atomic coordinates and isotropic or equivalent isotropic displacement parameters (Å^2^)

**Table:**

	*x*	*y*	*z*	*U*_iso_*/*U*_eq_	
Cl1	0.22510 (10)	0.32842 (19)	0.13842 (11)	0.0778 (5)	
N1	0.3173 (2)	0.2738 (6)	0.7453 (3)	0.0584 (9)	
O1	0.2028 (2)	0.1765 (5)	0.3854 (3)	0.0738 (10)	
O2	0.27033 (18)	0.3692 (4)	0.5103 (3)	0.0553 (8)	
O3	0.3734 (2)	0.3131 (6)	0.6813 (4)	0.0923 (13)	
O4	0.3268 (3)	0.1925 (6)	0.8386 (3)	0.0919 (13)	
O5	0.0483 (3)	0.3553 (6)	0.9720 (4)	0.0923 (13)	
C1	0.3298 (3)	0.3250 (5)	0.3259 (4)	0.0510 (11)	
C2	0.4090 (3)	0.3457 (6)	0.3744 (5)	0.0613 (12)	
H2	0.4163	0.3370	0.4552	0.074*	
C3	0.4757 (3)	0.3789 (7)	0.3028 (6)	0.0732 (14)	
H3	0.5280	0.3908	0.3352	0.088*	
C4	0.4654 (4)	0.3944 (7)	0.1839 (5)	0.0787 (17)	
H4	0.5109	0.4163	0.1362	0.094*	
C5	0.3891 (4)	0.3780 (7)	0.1347 (5)	0.0733 (15)	
H5	0.3827	0.3907	0.0540	0.088*	
C6	0.3205 (3)	0.3422 (6)	0.2056 (4)	0.0587 (12)	
C7	0.2598 (3)	0.2788 (7)	0.4038 (4)	0.0534 (11)	
C8	0.2101 (3)	0.3581 (6)	0.5953 (4)	0.0474 (10)	
C9	0.1291 (3)	0.4003 (7)	0.5704 (4)	0.0556 (11)	
H9	0.1135	0.4276	0.4938	0.067*	
C10	0.0713 (3)	0.4020 (6)	0.6588 (4)	0.0564 (11)	
H10	0.0166	0.4285	0.6411	0.068*	
C11	0.0937 (3)	0.3646 (5)	0.7739 (4)	0.0510 (10)	
C12	0.1744 (3)	0.3209 (5)	0.7998 (4)	0.0488 (10)	
H12	0.1897	0.2937	0.8766	0.059*	
C13	0.2326 (3)	0.3176 (5)	0.7108 (4)	0.0452 (10)	
C14	0.0319 (4)	0.3748 (8)	0.8689 (6)	0.0731 (14)	
H14	−0.029 (4)	0.423 (8)	0.840 (5)	0.098 (18)*	

### Atomic displacement parameters (Å^2^)

**Table:**

	*U*^11^	*U*^22^	*U*^33^	*U*^12^	*U*^13^	*U*^23^
Cl1	0.0968 (11)	0.0838 (8)	0.0528 (7)	0.0064 (7)	−0.0095 (8)	−0.0049 (6)
N1	0.051 (2)	0.076 (2)	0.048 (2)	0.003 (2)	−0.0027 (18)	−0.0013 (19)
O1	0.079 (2)	0.080 (2)	0.062 (2)	−0.018 (2)	0.0142 (18)	−0.0172 (16)
O2	0.0544 (18)	0.075 (2)	0.0363 (16)	−0.0051 (15)	0.0054 (13)	−0.0026 (14)
O3	0.047 (2)	0.156 (4)	0.074 (3)	−0.006 (2)	0.0005 (18)	0.027 (2)
O4	0.076 (3)	0.142 (4)	0.058 (2)	0.024 (2)	−0.0046 (18)	0.028 (2)
O5	0.093 (3)	0.128 (3)	0.056 (3)	0.002 (2)	0.027 (2)	0.0030 (19)
C1	0.062 (3)	0.050 (2)	0.041 (2)	0.004 (2)	0.007 (2)	−0.0024 (17)
C2	0.065 (3)	0.062 (3)	0.057 (3)	0.000 (2)	0.007 (2)	−0.001 (2)
C3	0.067 (4)	0.071 (3)	0.081 (4)	−0.005 (3)	0.013 (3)	−0.008 (2)
C4	0.087 (4)	0.072 (3)	0.077 (4)	−0.022 (3)	0.034 (3)	−0.015 (3)
C5	0.105 (5)	0.064 (3)	0.051 (3)	−0.008 (3)	0.028 (3)	−0.007 (2)
C6	0.073 (3)	0.054 (3)	0.050 (3)	0.004 (2)	0.003 (2)	−0.0065 (18)
C7	0.061 (3)	0.058 (3)	0.042 (3)	0.002 (2)	0.004 (2)	−0.0011 (19)
C8	0.049 (3)	0.051 (2)	0.043 (2)	−0.0021 (18)	0.0048 (18)	−0.0021 (16)
C9	0.054 (3)	0.067 (3)	0.047 (2)	0.006 (2)	−0.005 (2)	0.0046 (19)
C10	0.048 (2)	0.062 (2)	0.060 (3)	0.002 (2)	−0.004 (2)	−0.004 (2)
C11	0.048 (3)	0.053 (2)	0.051 (3)	−0.003 (2)	0.0101 (19)	−0.0063 (19)
C12	0.054 (3)	0.051 (2)	0.041 (2)	−0.0049 (19)	0.005 (2)	0.0009 (16)
C13	0.044 (2)	0.050 (2)	0.041 (2)	−0.0013 (18)	−0.0014 (19)	−0.0004 (16)
C14	0.061 (3)	0.082 (3)	0.076 (4)	−0.011 (3)	0.022 (3)	−0.011 (3)

### Geometric parameters (Å, º)

**Table:**

Cl1—C6	1.731 (5)	C4—C5	1.365 (8)
N1—O3	1.200 (5)	C4—H4	0.9300
N1—O4	1.221 (5)	C5—C6	1.400 (7)
N1—C13	1.464 (6)	C5—H5	0.9300
O1—C7	1.195 (6)	C8—C9	1.378 (6)
O2—C8	1.380 (5)	C8—C13	1.396 (6)
O2—C7	1.385 (6)	C9—C10	1.376 (6)
O5—C14	1.213 (7)	C9—H9	0.9300
C1—C6	1.385 (7)	C10—C11	1.388 (6)
C1—C2	1.408 (7)	C10—H10	0.9300
C1—C7	1.480 (6)	C11—C12	1.378 (6)
C2—C3	1.377 (7)	C11—C14	1.479 (7)
C2—H2	0.9300	C12—C13	1.387 (6)
C3—C4	1.370 (9)	C12—H12	0.9300
C3—H3	0.9300	C14—H14	1.10 (6)
			
O3—N1—O4	122.9 (4)	O1—C7—C1	128.7 (4)
O3—N1—C13	120.1 (4)	O2—C7—C1	109.1 (4)
O4—N1—C13	116.9 (4)	C9—C8—O2	121.3 (4)
C8—O2—C7	120.1 (3)	C9—C8—C13	119.3 (4)
C6—C1—C2	118.7 (5)	O2—C8—C13	119.3 (4)
C6—C1—C7	122.0 (4)	C10—C9—C8	120.1 (4)
C2—C1—C7	119.3 (4)	C10—C9—H9	120.0
C3—C2—C1	120.2 (5)	C8—C9—H9	120.0
C3—C2—H2	119.9	C9—C10—C11	120.8 (4)
C1—C2—H2	119.9	C9—C10—H10	119.6
C4—C3—C2	120.4 (6)	C11—C10—H10	119.6
C4—C3—H3	119.8	C12—C11—C10	119.7 (4)
C2—C3—H3	119.8	C12—C11—C14	120.0 (5)
C5—C4—C3	120.7 (5)	C10—C11—C14	120.3 (5)
C5—C4—H4	119.7	C11—C12—C13	119.6 (4)
C3—C4—H4	119.7	C11—C12—H12	120.2
C4—C5—C6	120.0 (5)	C13—C12—H12	120.2
C4—C5—H5	120.0	C12—C13—C8	120.6 (4)
C6—C5—H5	120.0	C12—C13—N1	116.6 (4)
C1—C6—C5	120.1 (5)	C8—C13—N1	122.9 (4)
C1—C6—Cl1	122.1 (4)	O5—C14—C11	123.7 (6)
C5—C6—Cl1	117.8 (4)	O5—C14—H14	122 (3)
O1—C7—O2	122.1 (4)	C11—C14—H14	114 (3)
			
C6—C1—C2—C3	−1.2 (6)	O2—C8—C9—C10	−175.0 (4)
C7—C1—C2—C3	176.6 (4)	C13—C8—C9—C10	−0.1 (6)
C1—C2—C3—C4	0.9 (7)	C8—C9—C10—C11	1.1 (6)
C2—C3—C4—C5	0.2 (8)	C9—C10—C11—C12	−1.6 (6)
C3—C4—C5—C6	−1.0 (8)	C9—C10—C11—C14	177.4 (4)
C2—C1—C6—C5	0.5 (6)	C10—C11—C12—C13	1.0 (6)
C7—C1—C6—C5	−177.2 (4)	C14—C11—C12—C13	−177.9 (4)
C2—C1—C6—Cl1	−177.3 (3)	C11—C12—C13—C8	−0.1 (6)
C7—C1—C6—Cl1	5.0 (6)	C11—C12—C13—N1	178.8 (3)
C4—C5—C6—C1	0.6 (7)	C9—C8—C13—C12	−0.4 (6)
C4—C5—C6—Cl1	178.5 (4)	O2—C8—C13—C12	174.6 (4)
C8—O2—C7—O1	−6.5 (7)	C9—C8—C13—N1	−179.2 (4)
C8—O2—C7—C1	175.7 (4)	O2—C8—C13—N1	−4.1 (6)
C6—C1—C7—O1	36.0 (7)	O3—N1—C13—C12	−161.5 (4)
C2—C1—C7—O1	−141.7 (5)	O4—N1—C13—C12	20.5 (6)
C6—C1—C7—O2	−146.4 (4)	O3—N1—C13—C8	17.3 (6)
C2—C1—C7—O2	35.9 (5)	O4—N1—C13—C8	−160.7 (4)
C7—O2—C8—C9	−51.7 (6)	C12—C11—C14—O5	3.7 (7)
C7—O2—C8—C13	133.4 (4)	C10—C11—C14—O5	−175.3 (5)

### Hydrogen-bond geometry (Å, º)

**Table:**

*D*—H···*A*	*D*—H	H···*A*	*D*···*A*	*D*—H···*A*
C14—H14···O4^i^	1.10 (6)	2.48 (6)	3.381 (7)	138 (4)

Symmetry code: (i) *x*−1/2, −*y*+1/2, *z*.
